# Nanocarrier Lipid Composition Modulates the Impact of Pulmonary Surfactant Protein B (SP-B) on Cellular Delivery of siRNA

**DOI:** 10.3390/pharmaceutics11090431

**Published:** 2019-08-23

**Authors:** Roberta Guagliardo, Pieterjan Merckx, Agata Zamborlin, Lynn De Backer, Mercedes Echaide, Jesus Pérez-Gil, Stefaan C. De Smedt, Koen Raemdonck

**Affiliations:** 1 Ghent Research Group on Nanomedicines, Laboratory of General Biochemistry and Physical Pharmacy, Department of Pharmaceutics, Ghent University, Ottergemsesteenweg 460, 9000 Ghent, Belgium; 2 Departamento de Bioquímica y Biología Molecular, Facultad de Biologia, and Research Institute Hospital 12 de Octubre, Universidad Complutense, José Antonio Novais 12, 28040 Madrid, Spain

**Keywords:** siRNA delivery, nanoparticles, pulmonary surfactant

## Abstract

Two decades since the discovery of the RNA interference (RNAi) pathway, we are now witnessing the approval of the first RNAi-based treatments with small interfering RNA (siRNA) drugs. Nevertheless, the widespread use of siRNA is limited by various extra- and intracellular barriers, requiring its encapsulation in a suitable (nanosized) delivery system. On the intracellular level, the endosomal membrane is a major barrier following endocytosis of siRNA-loaded nanoparticles in target cells and innovative materials to promote cytosolic siRNA delivery are highly sought after. We previously identified the endogenous lung surfactant protein B (SP-B) as siRNA delivery enhancer when reconstituted in (proteo) lipid-coated nanogels. It is known that the surface-active function of SP-B in the lung is influenced by the lipid composition of the lung surfactant. Here, we investigated the role of the lipid component on the siRNA delivery-promoting activity of SP-B proteolipid-coated nanogels in more detail. Our results clearly indicate that SP-B prefers fluid membranes with cholesterol not exceeding physiological levels. In addition, SP-B retains its activity in the presence of different classes of anionic lipids. In contrast, comparable fractions of SP-B did not promote the siRNA delivery potential of DOTAP:DOPE cationic liposomes. Finally, we demonstrate that the beneficial effect of lung surfactant on siRNA delivery is not limited to lung-related cell types, providing broader therapeutic opportunities in other tissues as well.

## 1. Introduction

Over the last two decades, research in the field of RNAi therapeutics has gained attention as it allows to address diseases at the transcriptome level [1]. Once they reached the cytosol, small interfering RNAs (siRNAs) activate the RNAi machinery, leading to post-transcriptional gene silencing through sequence-specific degradation of mRNA [2,3]. High target specificity and versatility of this emerging class of therapeutics represent some of the main advantages compared to conventional small molecule drugs and monoclonal antibodies, providing a wide range of biomedical uses [1,3]. However, their application in the clinic is limited by many extra- and intracellular delivery barriers. Most importantly, negatively charged hydrophilic macromolecules like siRNAs cannot cross biological membranes, making cellular delivery challenging [1,4].

Viral vectors are often applied carriers to guide cellular delivery of nucleic acids. However, labor-intensive large-scale production and safety issues remain important drawbacks, hence encouraging research for non-viral alternatives [4,5,6]. Encapsulation of siRNA into synthetic nanoparticles (NPs) allows its internalization by cells through endocytosis followed by release of the encapsulated RNA into the cytosol (i.e., endosomal escape). Among the vast number of NPs under investigation, cationic lipid nanoparticles (LNPs) currently are the preferred material for RNA delivery [7]. To date, many cationic lipid materials have been synthetized for LNP production [8,9,10,11,12,13]. However, the endosomal escape efficiency often remains poor [4,14,15,16]. Moreover, concerns remain regarding their safety and immunogenicity [12,17]. As such, to expedite clinical translation of this highly promising class of therapeutics, lipid-based siRNA formulations are needed to merge efficient cellular delivery with acceptable toxicity.

**Figure 1:**
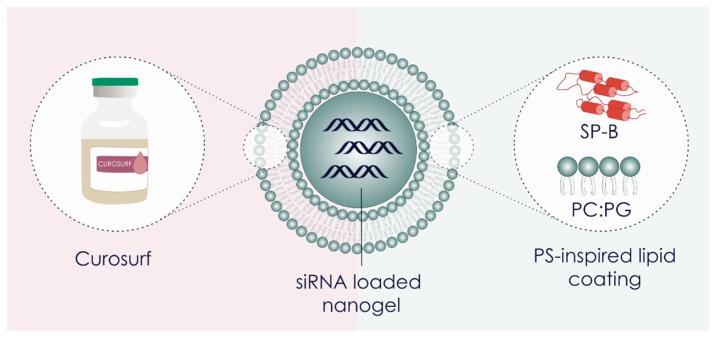
Visual representation of the core-shell surfactant-coated nanogel structure. SiRNA-loaded dextran nanogels (siNGs) were coated with Curosurf^®^ (poractant α; porcine derived clinical pulmonary surfactant (PS)) or with a PS-inspired lipid coating containing the surfactant protein B (SP-B) and an anionic lipid mixture. PC = phosphatidylcholine, PG = phosphatidylglycerol.

As synthetic polymer-and lipid-based NPs often fail to combine biocompatibility and efficacy, there is a growing interest in using bio-inspired materials [18]. We recently reported on a bio-inspired nanocomposite, composed of a siRNA-loaded polymeric matrix core surrounded by a shell of clinical pulmonary surfactant, i.e., poractant α (Curosurf^®^) (Figure 1) [19].

Pulmonary surfactant (PS) is a surface-active material that is produced and secreted into the alveolar space by specialized alveolar type II epithelial cells. PS covers the entire alveolar surface and its main physiological role is to maintain low surface tension upon expiration to prevent alveolar collapse [20]. Natural human PS has a complex composition of lipids (~90 wt%) and proteins (~10 wt%). The lipid fraction mainly contains zwitterionic phosphatidylcholine (PC) (~60–70 wt%) as well as anionic phosphatidylglycerol (PG) (~10 wt%) species and neutral lipids, of which cholesterol is the most abundant (~8–10 wt%). The protein fraction consists of two major classes of specialized surfactant proteins (SPs), i.e., the larger and hydrophilic SP-A and SP-D, as well as the smaller hydrophobic SP-B and SP-C [21,22]. PS has been extensively studied mainly because of its functional role in mammalian breathing [22,23]. In the context of inhalation therapy with nanomedicines, PS is primarily regarded as one of the extracellular barriers in the deep lung that needs to be overcome to gain access to underlying target cells upon inhalation therapy [21]. Its current therapeutic use is limited to the treatment of respiratory distress syndrome in premature infants, where modified PS from animal origin (e.g., Curosurf^®^) is approved for so-called surfactant replacement therapy [23].

Unexpectedly, we observed that the PS outer layer in the above mentioned nanocomposites significantly enhanced intracellular siRNA delivery in lung epithelial cells and (primary) alveolar macrophages [21,24]. Although it constitutes only a minor fraction in PS, surfactant protein B (SP-B) was identified as a key component for the improved RNA delivery (Figure 1) [25]. The beneficial effect of PS and the PS-associated SP-B on RNA delivery is a very recent finding and therefore remains largely unexplored in the literature. Qiu and colleagues reported on the cationic amphiphilic peptide KL4, a synthetic SP-B mimic, as siRNA carrier for lung delivery, reaching efficient delivery *in vitro* [26]. The Anderson group covalently conjugated the truncated cationic domain of SP-B to the surface of lipidoid NPs to improve siRNA delivery [27].

Hence, many questions remain on this unique function of PS and native SP-B. First, given its natural origin, proof-of-concept on SP-B promoted siRNA delivery has been limited to lung-related cell types. Here, we sought to confirm this specific activity of SP-B on other cell types as well. In addition, our earlier data suggest that the type of lipid with which the SP-B is associated, can influence its siRNA delivery efficiency. In this report, we therefore investigated the importance of the lipid composition on the siRNA delivery activity of SP-B in more detail. In particular, the impact of cholesterol, membrane fluidity and anionic lipid type in the SP-B inspired proteolipid shell of the nanocomposites is probed. Finally, we sought to reconstitute the cationic amphiphilic SP-B in DOTAP:DOPE cationic liposomes with the aim to promote their cellular siRNA delivery efficiency.

## 2. Materials and Methods

### 2.1. Small Interfering RNAs

Twenty-one nucleotide small interfering RNA (siRNA) duplexes targeting Enhanced Green Fluorescent Protein (siEGFP), non-targeting negative control duplexes (siCTRL), protein tyrosine phosphatase receptor type C (siCD45) and pGL3 firefly luciferase (siLuc) were purchased from Eurogentec (Seraing, Belgium). For cellular uptake experiments, the siCTRL duplex was labeled with a Cy5^®^ dye at the 5′ end of the sense strand (siCy5). The fluorescent labeling was performed and verified by Eurogentec. The concentration of the siRNA stock solutions in nuclease-free water (Ambion^®^-Life Technologies, Ghent, Belgium) was calculated from absorption measurements at 260 nm (1 OD260 = 40 μg/mL) with a NanoDrop 2000c UV-Vis spectrophotometer (Waltham, MA, USA). For siEGFP: sense strand = 5′-CAAGCUGACCCUGAAGUUCtt-3′; antisense strand = 5′-GAACUUCAGGGUCAGCUUGtt-3′. For siCTRL: sense strand = 5′-UGCGCUACGAUCGACGAUGtt-3′; antisense strand = 5′-CAUCGUCGAUCGUAGCGCAtt-3′. For siCD45: sense strand = 5′-GAA-GAA-UGC- UCA-CAG-AUA-A-3′; antisense strand = 5′-UUA-UCU-GUG-AGC-AUU-CUU-C-3′ (capital letters represent ribonucleotides; lower case letters represent 2′-deoxyribonucleotides). The sequence of siLuc is confidential and not available to be listed.

### 2.2. Synthesis of Dextran Nanogels and siRNA Complexation

Dextran hydroxyethyl methacrylate (dex-HEMA) or dextran methacrylate (dex-MA) [18,28,29,30] was copolymerized with a cationic methacrylate monomer [2-(methacryloyloxy)ethyl]- trimethyl-ammonium chloride (TMAEMA) to produce cationic dex-HEMA-*co*-TMAEMA (degree of substitution (DS) of 5.2) and dex-MA-*co*-TMAEMA (DS of 5.9) nanogels (hereafter abbreviated as respectively dex-HEMA NGs and dex-MA NGs), using an inverse miniemulsion photopolymerization method as previously reported [24,25,31,32,33]. The synthetized NGs were lyophilized and stored desiccated to ensure long term stability. To make siRNA-loaded nanogels (siNGs), 2 mg/mL of NG stock solutions were prepared by dispersing a weighed amount of the lyophilized nanoparticles in ice-cooled nuclease-free water, followed by brief sonication (Branson Ultrasonics Digital Sonifier^®^, Danbury, CT, USA). To allow siRNA complexation, equal volumes of siRNA and NGs in (4-(2-hydroxyethyl)-1-piperazineethanesulfonic acid) (HEPES) buffer (20 mM, pH 7.4) were mixed and incubated for ≥ 10 min at 4 °C.

### 2.3. Preparation of Proteolipid-Coated Nanogels

The commercially available clinical lung surfactant derived from minced porcine lungs, Poractant α (Curosurf^®^) (Chiesi Pharmaceuticals, Parma, Italy), was used to form the pulmonary surfactant (PS) outer layer on siNGs. To prepare the PS-inspired proteolipid coating the following lipids were used: 1,2-dioleoyl-*sn*-glycero-3-phosphocholine (DOPC), 1,2-dipalmitoyl-*sn*-glycero-3- phosphocholine (DPPC), 1,2-distearoyl-*sn*-glycero-3-phosphocholine (DSPC), l-α-phosphatidyl- glycerol from egg yolk (egg PG), l-α-phosphatidyl-l-serine (soy PS) and l-α-phosphatidylinositol from soy (soy PI). Soy PS was purchased from Sigma-Aldrich, all other lipids were obtained from Avanti Polar Lipids (Alabaster, AL, USA). SP-B was isolated from native porcine pulmonary surfactant following a procedure described earlier by Pérez-Gil and coworkers [34]. The lipids with or without SP-B (0.4 wt%) were mixed at the required weight ratios in chloroform and a (proteo) lipid film was obtained via nitrogen flow or rotary evaporation. The resulting lipid film was hydrated using HEPES buffer (20 mM, pH 7.4) and subsequently mixed with equal volumes of the previously formed siNGs (15 mg lipid/mg nanogel) [25]. The formation of the proteolipid coat was obtained by ≥ 10 min incubation at 4 °C and three 10″ cycles of high-energy sonication (amplitude 10%), using a probe sonicator (Branson Ultrasonics Digital Sonifier^®^, Danbury, CT, USA). To obtain Curosurf-coated siNGs (CS-NGs), the Curosurf^®^ dispersion (80 mg/mL) was diluted in HEPES buffer and mixed in equal volumes with the previously formed siNGs (15 mg lipid/mg nanogel), following an identical incubation and sonication protocol as detailed above. Hydrodynamic diameter, dispersity (Ð) and ζ-potential of all formulations were measured via Dynamic Light Scattering (DLS) (Zetasizer Nano, Malvern Instruments, Worcestershire, UK).

### 2.4. Preparation of Cationic Liposomes

To prepare cationic liposomes, 1,2-dipalmitoyl-3-trimethylammonium-propane (DOTAP) and 1,2-dioleoyl-*sn*-glycero-3-phosphoethanolamine (DOPE) were purchased from Avanti Polar Lipids (AL, USA). DOTAP:DOPE liposomes (50:50 molar ratio) were prepared by mixing appropriate amount of the mentioned lipids in chloroform in a round bottom flask. In the case of DOTAP:DOPE:SP-B liposomes, 1 wt% of SP-B was added to the lipid mixture in chloroform. A lipid film was obtained via rotary evaporation and subsequently hydrated with HEPES buffer (20 mM, pH 7.4). The lipid or lipid-protein dispersion was sonicated using a probe sonicator (Branson Ultrasonics Digital Sonifier^®^, Danbury, CT, USA) for 30″ via a pulsed program using 10% amplitude. DOTAP:DOPE liposomes were complexed with siRNA at a charge ratio (nitrogen/phosphate ratio) equal to 8, to obtain the formation of the so-called lipoplexes (LPX). Hydrodynamic diameter, dispersity (Ð) and ζ-potential of all formulations were measured via Dynamic Light Scattering (DLS) (Zetasizer Nano, Malvern Instruments, Worcestershire, UK).

### 2.5. Cell Lines and Culture Conditions

Cell culture experiments were performed using a human non-small cell lung cancer cell line stably expressing EGFP (H1299_eGFP) [25], a human ovarian cancer cell line stably expressing luciferase (SKOV3_LUC) [35], a human hepatoma cell line stably expressing eGFP (Huh-7_eGFP) [36] and a murine alveolar macrophage cell line (MH-S). The H1299_eGFP and SKOV-3_LUC were respectively obtained from the lab of Prof. Foged (Department of Pharmacy, University of Copenhagen, Copenhagen, Denmark) and the lab of Prof. Aigner (Institute of Pharmacology, Pharmacy and Toxicology, University of Leipzig, Leipzig, Germany). The Huh-7 cell line was obtained from the lab of Prof. Lahoutte, (VUB, Brussels, Belgium). Huh-7_eGFP were generated by transfecting Huh-7 cells with the pEGFP-N2 plasmid (Clontech, Palo Alto, CA, USA). The MH-S cell line was provided by VIB-UGent. H1299_eGFP cells were cultured in RPMI 1640, supplemented with 10% fetal bovine serum, 2 mM glutamine and 100 U/mL penicillin/streptomycin. Cells were treated with medium containing 1 mg/mL Geneticin^®^ once per month. SKOV-3_LUC were cultured in McCoy’s 5A medium, supplemented with 10% FBS and 100 U/mL penicillin/streptomycin. Huh-7_eGFP cells were cultured in DMEM:F12 supplemented with 10% fetal bovine serum, 2 mM glutamine and 100 U/mL penicillin/streptomycin. MH-S were cultured in RPMI 1640 supplemented with 2 mM glutamine, 10% fetal bovine serum, 100 U/mL penicillin/streptomycin, 10 mM HEPES, 1 mM sodium pyruvate and 0.05 mM 2-mercaptoethanol. All cells were cultured at 37 °C in a humidified atmosphere containing 5% CO_2_ and were passed every 3 days using a 0.25% trypsin-ethylenediaminetetraacetic acid (EDTA) solution to maintain subconfluency. All cell culture materials were purchased from Gibco^®^-Life Technologies, except for the serum, which was delivered by Hyclone™ (Thermo Fisher Scientific, Waltham, MA, USA).

### 2.6. Quantification of In Vitro Cellular siRNA Uptake by Flow Cytometry

To quantify the cellular internalization of siRNA via flow cytometry, H1299_eGFP cells (2 × 10^4^ cells/cm^2^ in 24-well plates), MH-S cells (4 × 10^4^ cells/cm^2^ in 12-well plates), Huh-7_eGFP cells (4 × 10^4^ cells/cm^2^ in 24-well plates) or SKOV-3_LUC cells (1.85 × 10^4^ cells/cm^2^ in 24-well plates) were plated (Greiner Bio-One GmbH, Kremsmünster, Austria) and allowed to settle overnight. NGs were loaded with siCTRL:siCy5 (100:0.75 mol%) and coated with a proteolipid mixture using the procedure described above. The particles were diluted 5 times in Opti-MEM to a final concentration of 30 µg/mL and incubated with the cells for 4 h (37 °C, 5% CO_2_). Next, the cells were washed with dextran sulfate sodium salt (0.1 mg/mL in PBS) to remove cell surface-bound fluorescence prior to flow cytometric quantification. To quantify uptake percentage, the mean fluorescence intensity (MFI) of cells treated with coated NGs were normalized to the ones of cells treated with uncoated NGs (representing 100%). For cationic liposomes, H1299_eGFP cells were seeded in 96-well plates (SPL Lifesciences Co. Ltd., Gyeonggi-do, South Korea) at a density of 2 × 10^4^ cells/cm^2^ and allowed to settle overnight. Liposomes were used for complexation of a mixture of siCTRL:siCy5 (90:10 mol%), diluted in Opti-MEM, and incubated with the cells for 4 h (37 °C, 5% CO_2_), followed by flow cytometric analysis as mentioned above. Data analysis was performed using the FlowJo^TM^ analysis software (Treestar, Costa Mesa, CA, USA).

### 2.7. Quantification of eGFP Gene Silencing by Flow Cytometry

To quantify gene knockdown efficiency, H1299_eGFP cells (2 × 10^4^ cells/cm^2^) or Huh-7_eGFP (4 × 10^4^ cells/cm^2^) were plated in 24-well plates (Greiner Bio-One GMBH) and allowed to settle overnight. Particles were prepared in Opti-MEM as described above and incubated with the cells (4 h at 37 °C and 5% CO_2_). Next, cells were washed with PBS and incubated with 1 mL fresh cell culture medium for 48 h. At this point, cells were prepared for flow cytometry as described above and a minimum of 10^4^ cells were analyzed for each sample. The eGFP expression percentage was calculated normalizing the MFI of cells treated with siEGFP to the MFI of cells treated with siCTRL. Data analysis was performed using the FlowJo^TM^ analysis software (Version 10.5.3, Treestar, Costa Mesa, CA, USA, 1997-2018).

### 2.8. Luciferase Silencing in Human Ovarian Carcinoma Cells

SKOV-3_LUC were seeded in 24-well plates at a density of 1.85 × 10^4^ cells/cm^2^ and allowed to attach overnight. Particles were prepared as described above and diluted in Opti-MEM before incubation with the cells (4 h at 37 °C and 5% CO_2_). Next, cells were washed with PBS and incubated with 1 mL fresh cell culture medium for 24 h. At this time point, cell culture medium was removed and the cells were washed with PBS. Subsequently, luminescence was measured using the Luciferase Reporter Assay Kit, following the optimized Promega protocols and reagents. Luciferase activity of each sample was assayed in a GloMax™ 96 Luminometer (Promega, Madison, WI, USA).

### 2.9. Quantification of In Vitro CD45 Silencing in MH-S by Flow Cytometry

MH-S cells were seeded in 12-well plates and allowed to settle overnight. Particles were prepared as described above and diluted in Opti-MEM (final NG concentration of 30 µg/mL; final siRNA concentration of 100 nM) before incubation with the cells (4 h at 37 °C and 5% CO_2_). Afterwards, the cells were washed with PBS and 1 mL of culture medium was added. Forty-eight hours after transfection, the cells were detached with a non-enzymatic cell dissociation buffer (10 min incubation at 37 °C). After centrifugation (7 min, 300 g), the cell pellet was resuspended in staining buffer (PBS supplemented with 5% FBS). High-affinity Fc receptors were blocked by incubation with purified anti-mouse CD16/CD32 (BD Biosciences, Erembodegem, Belgium) for 15 min at 4 °C. Subsequently, the cells were incubated with PerCP-Cy^®^ 5.5 rat anti-mouse CD45 (BD Biosciences) diluted in staining buffer and put on a rotary shaker for 45 min at room temperature for incubation. Following three washing steps with 1 mL staining buffer, the cell pellet was resuspended in 500 μL flow buffer and placed on ice until flow cytometry analysis. Subsequently cells were analyzed using a FACSCalibur^TM^ flow cytometer (BD Biosciences, Erembodegem, Belgium). The fluorescence for the Cy^®^ 5.5-label was measured at 488/690 nm. Data analysis was performed using the FlowJo™ analysis software (Treestar, Costa Mesa, USA).

### 2.10. Statistical Analysis

All experiments were performed in technical triplicate and with ≥ 2 independent biological repeats (≥ *n* = 2), unless otherwise stated. All data are presented as mean ± standard deviation (SD). Statistical analysis was performed via one way ANOVA, unless otherwise stated, followed by a Bonferroni multiple comparison test, using GraphPad Prism software version 8.

## 3. Results and Discussion

### 3.1. Pulmonary Surfactant (PS) Potentiates siRNA Delivery in Non-Pulmonary Cell Lines

**Figure 2:**
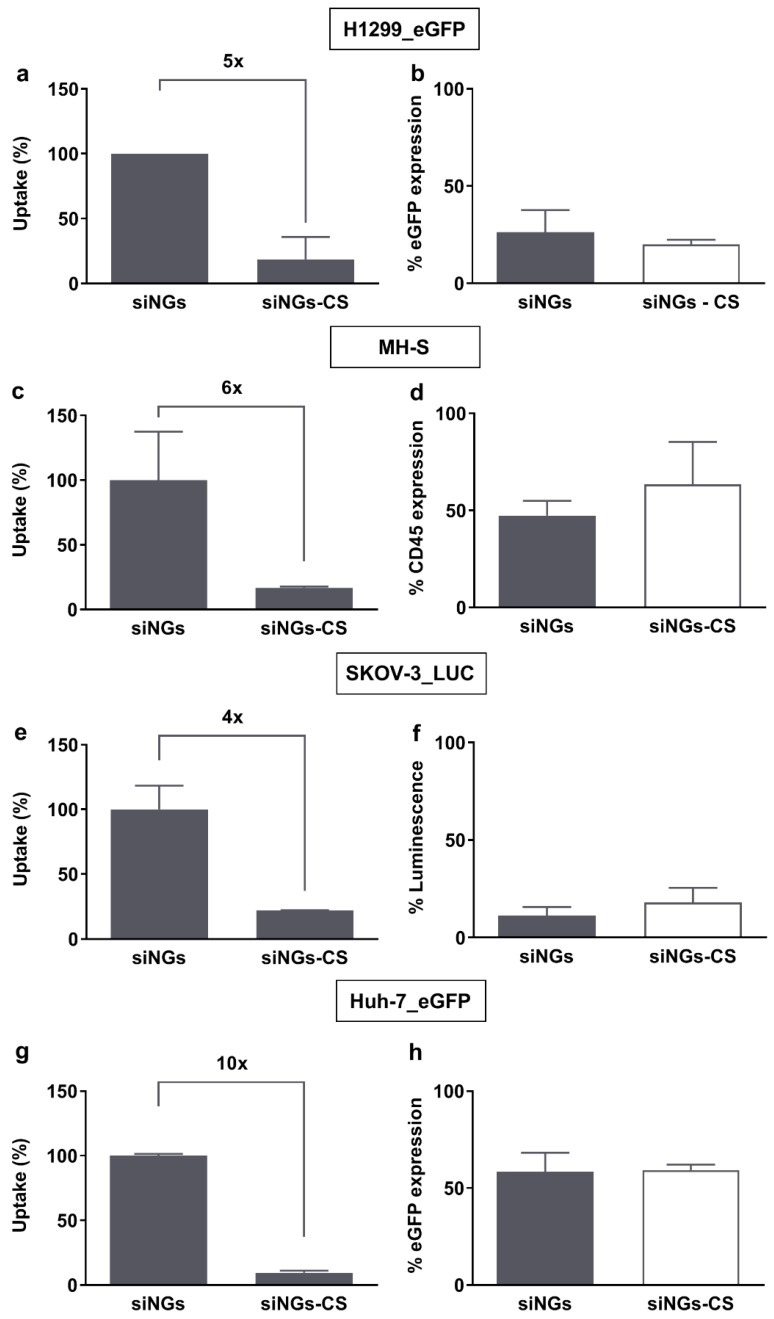
Biological efficacy of surfactant-coated nanogels on (non-)pulmonary cell lines. (**a,c,e,g**) Flow cytometric quantification of cellular uptake of siCy5-loaded nanogels (siNGs) with and without Curosurf^®^ (CS) coating. (**b,d,f,h**) Gene silencing potential of siNGs and Curosurf^®^- coated NGs (siNGs-CS). Despite the strongly reduced cellular uptake of siNGs following Curosurf^®^ coating, both formulations reach comparable levels of gene knockdown on the different cell lines studied. Experiments were performed with a fixed NG concentration (30 µg/mL) and siRNA concentration (50 nM), except for the MH-S cell line, for which we used a final siRNA concentration of 100 nM. Experiments on H1299_eGFP and silencing of MH-S cell lines are the result of three independent biological repeats (*n* = 3), other experiments are performed in technical triplicate.

As mentioned above, earlier work has demonstrated improved siRNA delivery and targeted gene silencing with PS-coated nanocomposites in both non-small cell lung cancer cells (H1299) and alveolar macrophages [21,24,25]. Corroborating these results, as shown in Figure 2a, layering cationic siNGs with a negatively charged PS bilayer (i.e., Curosurf^®^) strongly reduces cellular uptake in the H1299 cell line. Importantly, despite the lower intracellular siRNA dose, the same level of targeted gene knockdown is obtained (Figure 2b), indicating that the CS coat enhances the fraction of the internalized siRNA dose that is delivered into the cytosol. A comparable outcome was obtained for the murine alveolar macrophage cell line MH-S, targeting the CD45 gene (Figure 2c,d). To evaluate if PS can likewise promote siRNA delivery in cell lines derived from other organs, human ovarian carcinoma cells (SKOV-3) and human hepatoma cells (Huh-7) were treated with CS-coated siNGs (Figure 2e–h). Consistent with earlier reports, the anionic CS outer layer significantly inhibited cellular internalization of siNGs in both cell types. However, despite the ≥4-fold reduction in intracellular siRNA dose, also in these cell lines a comparable knockdown of the targeted reporter genes relative to the uncoated siNGs was observed, albeit that the Huh-7 reporter cell line in general appeared to be more difficult to transfect. These data support the notion that although the lungs constitute the natural habitat of lung surfactant, its beneficial effect on intracellular siRNA delivery is not limited to lung-related cell types and that PS-inspired drug delivery should not be restricted to the lungs as main target tissue.

### 3.2. The Activity of SP-B Is Dependent on Its Lipid Microenvironment

**Figure 3:**
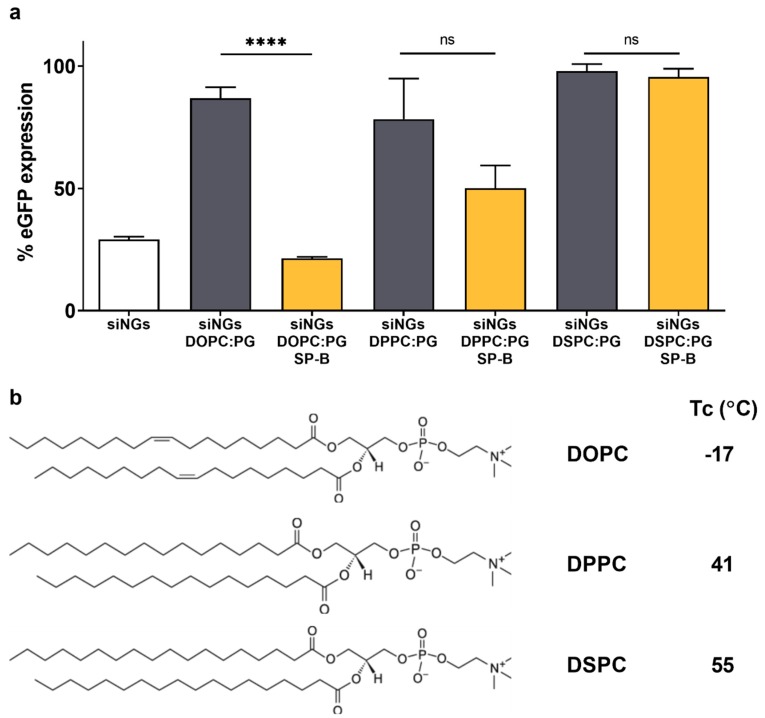
Impact of lipid phase transition temperature on formation and delivery efficiency of SP-B containing proteolipid-coated nanogels. (**a**) Evaluation of eGFP silencing in H1299_eGFP cells by uncoated or (proteo)lipid-coated siNGs with different lipid mixtures, supplemented with 0.4 wt% SP-B. All experiments were performed with a fixed NG concentration (30 µg/mL) and siRNA concentration (50 nM). The SP-B effect is strongly influenced by the type of lipid with which it is combined, highlighting the importance of a fluid lipid membrane in the formulation of the core-shell nanocomposites. (**b**) Chemical structures and phase transition temperatures (Tc) of the different PC lipids tested. Statistical analysis was performed via an unpaired *t*-test. Data are represented as the mean ± SD (*n* = 2) and statistical significance is indicated (**** *p* < 0.0001, ns = not significant).

In recent work, Merckx et al. revealed that surfactant protein B (SP-B) is a key component in lung surfactant that dictates cellular siRNA delivery [25]. However, the activity of SP-B was proven to be strongly dependent on the type of lipids with which it is combined, with the more fluid lipid mixture DOPC:PG (85:15 wt%) clearly outperforming its more rigid counterpart DPPC:PG (85:15 wt%) in terms of *in vitro* siRNA delivery efficiency. It is postulated that less resistance against lateral movement in a less rigid proteolipid coat could promote SP-B-mediated intermembrane interactions [34,37]. To extend our understanding, siNGs were coated with DSPC:PG (85:15 wt%) of which the main lipid has a substantially higher phase transition temperature (Tc) of 55 °C, compared to DPPC (41 °C). The absence of SP-B in the lipid coat inhibited siNG-mediated gene silencing, independent of the type of lipid used. Supplementation of the DOPC:PG and DPPC:PG lipid coat with SP-B did result in reduced eGFP expression levels, albeit that the improvement in gene silencing was statistically significant solely for the more fluid DOPC-containing lipid bilayer (Figure 3a). Importantly, with DSPC as main phospholipid, no gene silencing could be observed anymore. Of note, using an identical coating protocol, the use of DSPC as main lipid component resulted in micrometer sized nanocomposites (Table 1). These data altogether suggest that the Tc of the lipid coat influences both the colloidal stability of the core-shell formulation as well as its intracellular siRNA delivery potential.

Nanogels (NGs); Dextran hydroxyethyl methacrylate (dex-HEMA); dextran methacrylate (dex-MA); lipid coating DOPC:PG (LIP); Surfactant Protein B (SP-B); lipoplexes (LPX); polydispersity (Ð). Samples were measured in HEPES buffer 20 mM pH 7.4.

**Figure 4:**
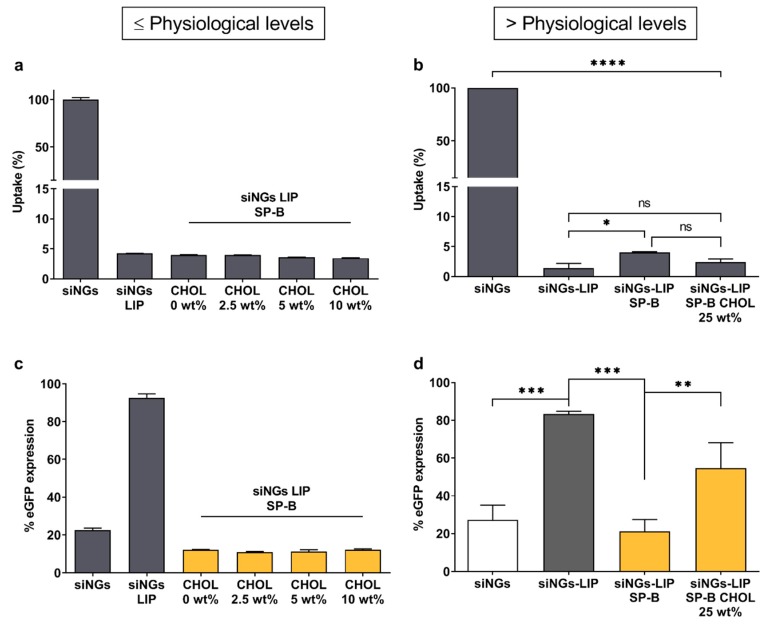
Impact of cholesterol on biological efficacy of proteolipid-coated nanogels. Evaluation of (**a**) cellular uptake and (**c**) gene silencing potential in H1299_eGFP cells of siRNA-loaded nanogels (siNGs) coated with lipid mixtures containing physiological cholesterol (CHOL) levels (2.5, 5 to 10 wt%). Data show one representative (technical triplicate) of two independent experiments; formulations with different siRNA concentrations showed the same trend (data not shown). Evaluation of (**b**) cellular uptake and (**d**) gene silencing potential of siNGs coated with increased cholesterol fraction in the outer layer (~25 wt%) (*n* = 3). Cholesterol exceeding physiological levels partially hinder SP-B promoted siRNA delivery. All experiments were performed with a fixed NG concentration (30 µg/mL) and siRNA concentration (50 nM). LIP = DOPC:PG (85:15); LIP SP-B = DOPC:PG (85:15) + SP-B 0.4 wt%; LIP SP-B CHOL = DOPC:CHOL:PG (60:25:15) + SP-B 0.4 wt%. Data are represented as the mean ± SD and statistical significance is indicated (* *p* < 0.05, ** *p* < 0.01, *** *p* < 0.005, **** *p* < 0.0001, ns = not significant).

Natural PS contains a substantial fraction of neutral lipids, mainly cholesterol (~8 wt%), which modulate surfactant activity [38]. However, excessive cholesterol levels are known to interfere with normal surfactant function, possibly contributing to respiratory insufficiency [39,40]. As the manufacturing of Curosurf^®^ involves depletion of neutral lipids, including cholesterol and cholesteryl esters, the data shown in Figure 2 and in earlier reports seem to indicate that the presence of cholesterol is not strictly required for the siRNA delivery-promoting effect of SP-B. However, state-of-the-art lipid formulations often contain high fractions of cholesterol (up to 40 wt%) as a stabilizing component for *in vivo* application. Therefore, we sought to probe the impact of cholesterol on SP-B mediated siRNA delivery (Figure 4). While supplementation of the DOPC:eggPG lipid mixture with physiological cholesterol levels did not negatively influence siRNA delivery efficiency, gene silencing efficiency was slightly impaired when further increasing the cholesterol fraction to ~25 wt%. Overall, we conclude that cholesterol levels exceeding the endogenous PS fractions by far, partially hamper SP-B’s beneficial effect on siRNA delivery.

**Figure 5:**
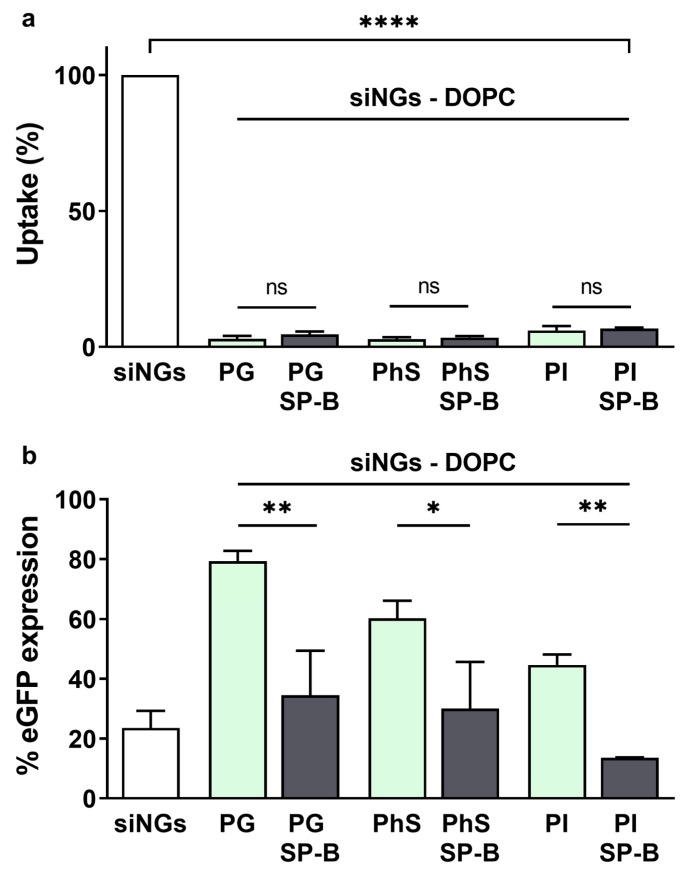
Role of the anionic lipid in biological efficacy of proteolipid-coated nanogels. (**a**) Cellular uptake and (**b**) gene silencing evaluated on H1299_eGFP cells via flow cytometry. SiRNA-loaded nanogels (siNGs) were coated with a mixture of DOPC:PG, DOPC:PhS or DOPC:PI (weight ratio 85:15). The presence of negatively charged lipids is required to allow the formation of the core-shell structure via electrostatic interactions. The replacement of the anionic phosphatidylglycerol (PG) with phosphatidylserine (PhS) or phosphatidylinositol (PI) does not abrogate SP-B’s beneficial effect on siRNA delivery. All experiments were performed with a fixed NG concentration (30 µg/mL) and siRNA concentration (50 nM). Statistical analysis was performed via an unpaired *t*-test. Data are represented as the mean ± SD (*n* = 3) and statistical significance is indicated (* *p* < 0.05, ** *p* < 0.01, **** *p* < 0.0001, ns = not significant).

While anionic phospholipids are generally present in rather low concentrations in mammalian tissues, PS represents an exception with its PG content of 7–12% by mass [41]. As mentioned above, SP-B is a cationic amphipathic protein, of which the positive charges are believed to interact with the head groups of anionic phospholipid species like PG. As previously reported, this interaction could orchestrate the distribution of SP-B in the more disordered phases of PS membranes [42,43]. It is conceivable that SP-B likewise connects electrostatically with the PG fraction in the nanocomposite proteolipid coat, mimicking the natural interaction of SP-B with lipid bilayers. However, PG does not constitute the only negative phospholipid in PS, where phosphatidylinositol (PI) and phosphatidylserine (PhS) also play a role. Of note, PI represent the main negative phospholipid in lung surfactant of other species, while in humans only fetal surfactant shows higher PI content relative to PG, which is reversed with ageing [41]. The less abundant PhS seems to be mainly involved in surfactant metabolism processes, although its exact role is still unclear [41]. Here, we aimed to evaluate the compatibility of SP-B with other anionic lipids by replacing the PG fraction with PhS or PI in the SP-B supplemented DOPC:PG proteolipid coat of the nanocomposites (Figure 5). As expected, all coated formulations substantially reduced the cellular uptake of the siNGs (>10-fold). PI containing nanocomposites reached the highest knockdown levels, which could in part be explained by the relatively higher intracellular siRNA dose. Most importantly, independent of the type of anionic lipid, the presence of SP-B in the lipid coat significantly promotes gene silencing efficiency, indicating that the nature of the negative phospholipid is not critical for the cellular effect of SP-B (Figure 5).

### 3.3. Degradability of the Nanogel Core Does Not Influence SP-B Activity

**Figure 6:**
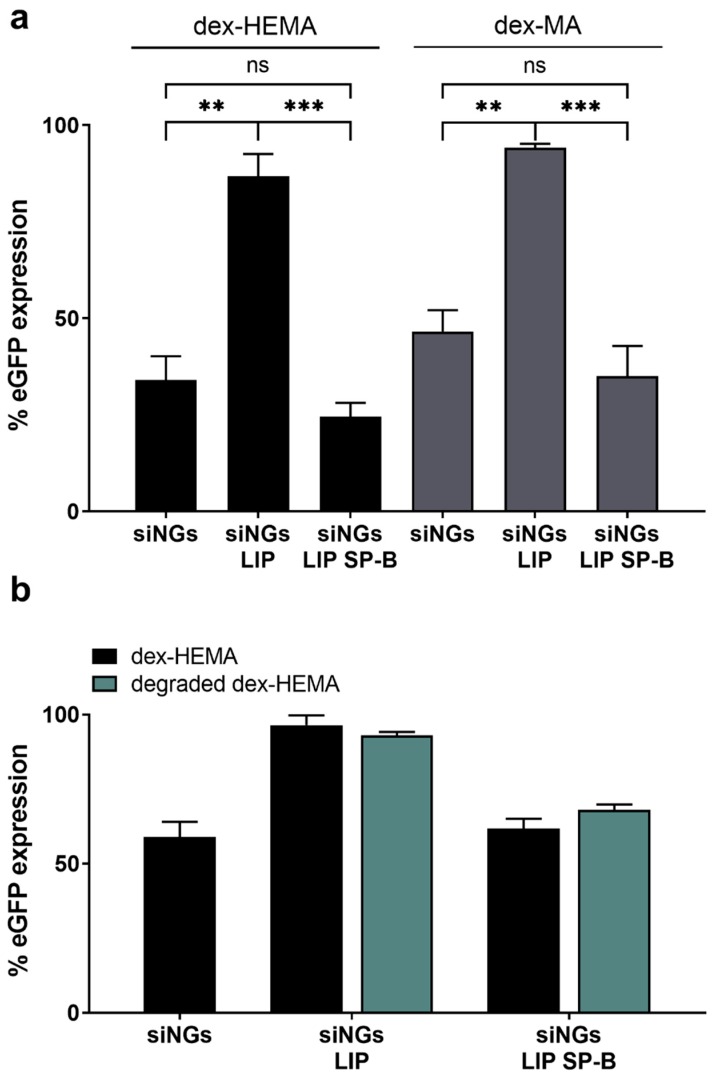
Impact of dextran nanogel core structure on SP-B mediated siRNA delivery. (**a**) Gene silencing potential of (proteolipid-coated) siRNA-loaded NGs (siNGs) constructed with the hydrolysable dex-HEMA or the stable dex-MA. Both formulations were coated with a mixture of DOPC:PG (85:15 wt%) here abbreviated as LIP, with or without SP-B. Although the stable dex-MA shows a less pronounced eGFP knockdown, SP-B promotes siRNA delivery equal to the degradable dex-HEMA. Data are a summary of two independent experiments. Data are represented as the mean ± SD (*n* = 2) and statistical significance is indicated (** *p* < 0.01, *** *p* < 0.005, ns = not significant). (**b**) Gene silencing of (proteo)lipid-coated dex-HEMA NGs with an intact or degraded NG core. All experiments were performed with a fixed NG concentration (30 µg/mL) and siRNA concentration (5 nM). Data show one representative graph of two independent experiments; formulations with increased SP-B fraction showed the same trend (data not shown).

To date, the effect of SP-B on siRNA delivery has only been demonstrated using PS-inspired proteolipid nanocomposites with a biodegradable hydrogel core (Figure 1). To evaluate whether the degradation of the core contributes to the activity of SP-B, the hydrolysable dex-HEMA NG core was replaced by its stable dex-MA counterpart with comparable physicochemical characteristics (Figure 6) [28,29,30]. In line with earlier data from our group [31,44], dex-MA siNGs show a slightly reduced intrinsic siRNA delivery potential relative to dex-HEMA siNGs (due to the absence of the hydrolysable carbonate ester in the crosslinks), albeit similar uptake levels are achieved (data not shown). However, coating of the former with a SP-B proteolipid bilayer also strongly promoted siRNA delivery and target gene knockdown, indicating that degradability of the core material is not essential for SP-B’s activity (Figure 6a).

In addition, intentionally degrading the dex-HEMA NG core (4 h incubation at 37 °C) after proteolipid coating but prior to transfection did not seem to affect the gene knockdown efficiency (Figure 6b). These results indicate that an intact polymeric core material in the core-shell nanocomposites is likewise not essential to the delivery-promoting effect of SP-B.

### 3.4. Integration of SP-B into Cationic Liposomes Does Not Enhance siRNA Delivery

**Figure 7:**
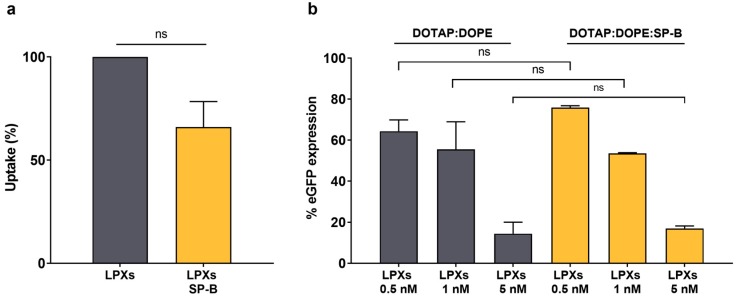
Evaluation of SP-B effect on DOTAP:DOPE liposomes for siRNA delivery in H1299_eGFP cells. (**a**) Cellular uptake and (**b**) gene silencing potential of DOTAP:DOPE LPXs (final concentrations siRNA are 0.5, 1 and 5 nM) with and without SP-B (1 wt%). The inclusion of SP-B in the cationic liposomal formulation does not result in any enhanced delivery effect. For uptake data, statistical analysis was performed using a one sample *t*-test. Data are represented as the mean ± SD (*n* = 2) and statistical significance is indicated (ns = not significant).

To form a stable core-shell nanocomposite, electrostatic interaction of a negatively charged lipid shell with the cationic NG core is required [19,24]. Here, the NG enables siRNA encapsulation in the nanocomposite and at the same time serves as a solid support for the deposition of the surfactant shell. However, as the integrity of the core material is not essential for SP-B’s effect on siRNA delivery, we aimed to evaluate the impact of SP-B supplementation in cationic liposomes in which the siRNA complexation is directly achieved by the positively-charged lipids. To date, cationic lipid nanoparticles (LNPs) remain the most advanced nanoformulation for siRNA delivery [45]. LNPs are typically composed of a cationic lipid and one or more helper lipids, in which the cationic lipid is the dominant component as it enables both electrostatic complexation of the oppositely charged RNA as well as cellular delivery by facilitating cellular uptake and endosomal escape, albeit that the latter step in general lacks efficiency for the majority of siRNA nanomedicines [46]. Increasing the cytosolic delivery potential of such formulations could reduce the dose of both carrier and cargo, thus mitigating the risk of off-target effects. Here, we aimed to exploit the amphiphilic properties of SP-B to reconstitute SP-B in the bilayer of a commercially available cationic liposomal formulation (i.e., DOTAP:DOPE 50:50 mol%). To obtain the lipoplexes (LPX), the formed liposomes were incubated with siRNA solutions to obtain a charge ratio equal to 8 [47]. Adding 1 wt% of SP-B to the lipid composition seemed to slightly reduce both hydrodynamic diameter as well as surface charge (Table 1). Likewise, also the cellular internalization in the H1299 cell model was decreased when SP-B was present in the DOTAP:DOPE bilayer, albeit without reaching statistical significance. Most importantly, although higher SP-B fractions were applied here, no beneficial effect on intracellular siRNA delivery and resulting target gene silencing could be noted in the presence of SP-B (Figure 7). It is hypothesized that the high intrinsic cationic charge density of the DOTAP:DOPE liposomes might obscure the more subtle membrane destabilizing effects of the cationic amphiphilic SP-B protein. On the other hand, it cannot be excluded that electrostatic repulsion between cationic lipids and positively-charged SP-B molecules could result in a less optimal distribution of the protein to enable intracellular siRNA delivery. More detailed experiments are required to fully elucidate the contrasting effects of SP-B when reconstituted in DOTAP:DOPE cationic liposomes.

## 4. Conclusions

Previous studies have identified the endogenous surfactant protein B (SP-B) as siRNA delivery enhancer when reconstituted in lipid-coated nanogels (NGs). The mechanism of action of SP-B at the alveolar air-liquid interface has been investigated in detail, providing essential knowledge of the impact of the lipid microenvironment on SP-B’s activity and, consequently, on surfactant dynamics. Contrarily, the way SP-B is able to promote intracellular delivery of siRNA and how this activity might be influenced by the lipid environment has not yet been described in detail. Here, we evaluated the influence of the main constituents of SP-B proteolipid-coated NGs, namely the lipid composition of the proteolipid coat and the degradability of the NG core, on the activity of SP-B. While the inner core degradability did not seem to be essential for SP-B promoted siRNA delivery, we showed a crucial role of the surrounding lipid membranes. Specifically, we described the importance of membrane fluidity and appropriate cholesterol levels, in analogy with the physiological interactions between SP-B and lipids occurring at the alveolar air-liquid interface. In addition, the adjuvant effect of SP-B on cellular siRNA delivery was supported by different types of anionic lipid species in the proteolipid coat. On the other hand, a formulation of commonly used DOTAP:DOPE cationic liposomes with SP-B did not result in an improved gene silencing effect. Importantly, we also showed the SP-B promoted siRNA delivery in other pulmonary cell lines, suggesting its suitability to boost siRNA delivery in extrapulmonary tissues as well. Altogether, these results provide useful insights that will support future rational design of lipid-based SP-B nanoplatforms for siRNA delivery.

## Figures and Tables

**Table 1:** Representative DLS data and ζ-potential of different formulations used in this study.

Sample	Hydrodynamic Diameter (nm)	Ð	ζ -Potential (mV)
NGs DOPC:PG	168 ± 2	0.24	−23 ± 1
NGs DPPC:PG	257 ± 2	0.45	−20 ± 1
NGs DSPC:PG	5480 ± 2700	1	−16 ± 0
Dex-HEMA NGs	195 ± 3	0.18	17 ± 0
Dex-HEMA NGs LIP	160 ± 1	0.24	−29 ± 1
Dex-HEMA NGs LIP SP-B	158 ± 3	0.26	−35 ± 0
Dex-MA NGs	203 ± 1	0.26	15 ± 3
Dex-MA NGs LIP	117 ± 1	0.26	−35 ± 0
Dex-MA NGs LIP SP-B	114 ± 2	0.39	−40 ± 1
DOTAP:DOPE LPX	132 ± 3	0.40	56 ± 4
DOTAP:DOPE LPX SP-B	114 ± 2	0.38	48 ± 2
